# A Comprehensive Review of Honey-Containing Hydrogel for Wound Healing Applications

**DOI:** 10.3390/gels11030194

**Published:** 2025-03-12

**Authors:** Andik Nisa Zahra Zainuddin, Nurul Nadhirah Mustakim, Farah Alea Rosemanzailani, Nur Izzah Md Fadilah, Manira Maarof, Mh Busra Fauzi

**Affiliations:** 1 Department of Tissue Engineering and Regenerative Medicine, Faculty of Medicine, Universiti Kebangsaan Malaysia, Jalan Yaacob Latif, Cheras, Kuala Lumpur 56000, Malaysia; p153345@siswa.ukm.edu.my (A.N.Z.Z.); nurul.nadhira68@gmail.com (N.N.M.); farah.alea10@gmail.com (F.A.R.); izzahfadilah@ukm.edu.my (N.I.M.F.); manira@ukm.edu.my (M.M.); 2 Advance Bioactive Materials-Cells UKM Research Group, Universiti Kebangsaan Malaysia, Bangi 43600, Selangor, Malaysia; 3 Ageing and Degenerative Disease UKM Research Group, Universiti Kebangsaan Malaysia, Bangi 43600, Selangor, Malaysia

**Keywords:** honey, hydrogel, in vitro, in vivo, honey wound healing mechanisms

## Abstract

Honey has long been recognized for its medicinal properties, particularly in wound healing. Recent advancements in material science have led to the development of honey-containing hydrogels, combining the natural healing properties of honey with the versatile characteristics of hydrogel matrices. These hydrogels offer numerous advantages, including high moisture retention, biocompatibility, and the controlled release of bioactive compounds, making them highly effective for wound healing applications. Hydrogels hold significant potential in advancing medical applications, particularly for cutaneous injuries. The diverse properties of honey, including antimicrobial, anti-inflammatory, and anti-eschar effects, have shown promise in accelerating tissue regeneration. According to studies, they are effective in maintaining a good swelling ratio index, Water Vapour Transmission Rate (WVTR), contact angle, tensile and elongation at break, in vitro biodegradation rate, viscosity and porosity analysis, lowering bacterial infections, and encouraging rapid tissue regeneration with notable FTIR peaks and SEM average pore sizes. However, limitations such as low bioavailability and inefficiencies in direct application reduce their therapeutic effectiveness at the wound site. Integrating honey into hydrogels can help preserve its wound healing mechanisms while enhancing its ability to facilitate skin tissue recovery. This review explores the underlying mechanisms of honey in wound healing management and presents an extensive analysis of honey-containing hydrogels reported in the literature over the past eight years. It emphasizes the physicochemical and mechanical effectiveness and advancements of honey-incorporated hydrogels in promoting skin wound healing and tissue regeneration, supported by evidence from both in vitro and in vivo studies. While honey-based therapies for wound healing have demonstrated promising outcomes in numerous in vitro and animal studies, clinical studies remain limited. Despite that, honey’s incorporation into hydrogel systems, however, offers a potent fusion of contemporary material technology and natural healing qualities, marking a substantial breakthrough in wound treatment.

## 1. Introduction

**Figure 1:**
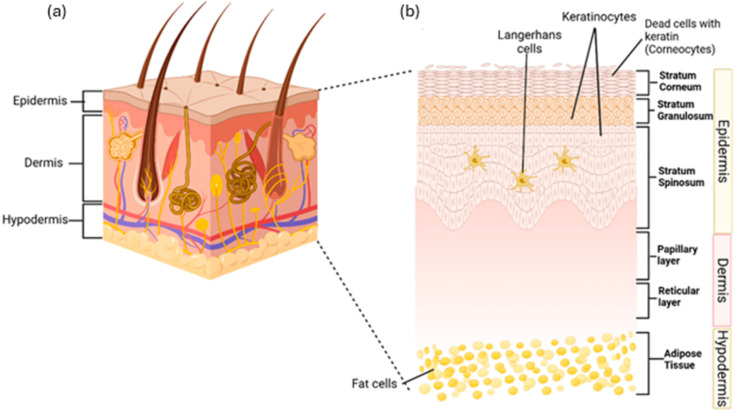
(**a**) Diagram of skin anatomy, showing the outer epidermis, middle dermis, and inner hypodermis. (**b**) The skin layer, the epidermis, consists of the stratum corneum with corneocytes, the stratum granulosum with live keratinocytes, and the stratum spinosum with live keratinocytes and Langerhans cells. The dermis includes the papillary layer, housing collagen, fibroblasts, and phagocytes, and the reticular layer, which contains elastin fibres. The hypodermis, located beneath the dermis, connects the skin to underlying bones and muscles. It consists of adipose tissue composed of fat cells. The idea of this figure is adapted from Gounden et al. (2024) [5].

The skin protects the body by acting as a primary physical barrier against physical harm, chemicals, germs, and fluid loss. It also plays a role in hydration, vitamin D production, temperature control, waste removal, and immune functions to maintain body homeostasis [1,2]. The skin has three main layers: the epidermis, dermis, and subcutaneous tissue [3]. The epidermis, the outer layer, serves as a barrier and contains sweat glands, sebaceous glands, and hair follicles. Beneath it, the dermis contains blood vessels, the extracellular matrix, and sensors, providing strength, nutrients, and immunity. The subcutaneous layer, the innermost part, consists of fat tissue that stores energy and supplies growth factors for dermis. All three layers also contain immune cells that monitor and respond to damage [4]. Figure 1a shows the anatomy of human skin while Figure 1b shows the layers of the skin in details.

**Figure 2:**
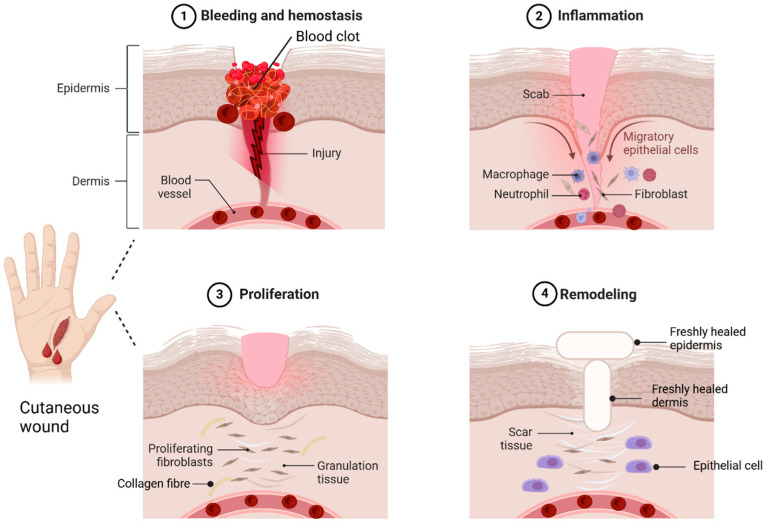
Wound healing stages. Haemostasis is the body’s first response to tissue damage, starting with vasoconstriction to reduce blood loss. The inflammation phase triggers innate and adaptive immune responses, facilitating neutrophil and macrophage migration to the injury site. The third phase, the proliferation phase, is dominated by fibroblasts, which release cytokines and collagen, leading to the formation of granulation tissue. In the remodelling phase, the ECM is restructured to resemble healthy tissue, leading to scar formation. This process is primarily regulated by myofibroblasts and epithelial cells. The idea of this figure is adapted from Deng et al. (2022) [20].

A skin wound is an injury or breakdown of skin tissue caused by trauma, thermal damage, or chronic conditions like pressure ulcers, venous stasis, or diabetes mellitus [6]. Skin wounds are classified as acute or chronic [7]. Acute wounds heal through a series of molecular steps that restore the skin’s structure, while chronic wounds fail to heal properly, leading to necrosis, infections, and prolonged inflammation [8]. Wound healing occurs in four phases: haemostasis, inflammation, proliferation, and remodelling [9]. In haemostasis, vasoconstriction and platelet aggregation at the injury site form a clot with proteins like fibronectin and collagen, which then converts fibrinogen into fibrin to seal the wound [9,10,11]. The inflammation phase involves neutrophils and macrophages migrating to the wound site. Neutrophils clear debris and pathogens through reactive oxygen species (ROS) and proteases [11]. Macrophages support tissue regeneration, release cytokines to amplify immune responses, recruit leukocytes, and clear apoptotic cells [12,13]. In the proliferation stage, fibroblasts release cytokines and collagen for repair, while platelets and white blood cells assist in cell migration [14]. This phase also involves new blood vessels, collagen production, and granulation tissue with epithelial cells, fibroblasts, and keratinocytes [15]. Remodelling, led by myofibroblasts, restores the extracellular matrix ECM, strengthens collagen, and reduces scar thickness as the wound closes [13,16,17]. Although wound healing is often successful, wounds remain a significant healthcare challenge. The global wound care market, valued at $18.4 billion in 2018, is projected to grow at a 3.9% compound annual growth rate (CAGR) from 2019 to 2026 [18]. Hence, successful wound healing relies on maintaining optimal microenvironmental conditions like proper moisture, pH balance, and oxygen levels [19]. Figure 2 shows the sequential steps of wound healing stages.

Common current wound treatments include dressings and surgery. Traditional dressings like gauze absorb moisture, drying the wound, slowing healing, and causing pain during removal [21]. The long-term outcomes of surgical interventions depend on bed rest duration, preoperative risk factors, and the potential for bacterial infections [5]. Therefore, it is crucial to identify new therapeutic targets and develop more effective treatment strategies to address these challenges. Recent years have seen a shift toward biomaterial scaffolds in wound management [22]. These three-dimensional (3D) porous structures support cell attachment, influencing proliferation and differentiation to aid tissue repair and regeneration [23]. A biomaterial is any material designed to interact with biological systems, ranging from inert implants to biologically integrated grafts. Biomaterials can be either naturally derived or polymers and lab-engineered agents with bioactive properties [24,25]. In general, two-dimensional (2D) scaffolds are formed as films, membranes, or fibres, while 3D scaffolds with porous structures come as sponges, foams, or hydrogels [26]. Among these, hydrogels are highly promising for wound healing due to their versatility and unique properties. These polymeric materials, formed through the crosslinking of hydrophilic polymer chains, have a three-dimensional structure with high water content. This maintains a moist environment, reducing scarring, providing a cooling effect, and minimizing tissue adherence to ease discomfort. Their adaptability allows hydrogels to cover wounds of irregular shapes effectively [5,15,27]. The use of natural products like honey dates back centuries, with ancient Egyptians applying it to wounds—a practice proven to promote healing and prevent infections [28].

Recent advancements in biotechnology have revealed new, unique components and medicinal properties in honey. Honey is rich in proteins, carbohydrates, moisture, enzymes, vitamins, minerals, phenols, and bioactive compounds like carotenoids, proline, flavonoids, salicylic acid, naringin, and taxifolin. It typically contains 80% carbohydrates and 20% water [29]. Honey has shown superior effectiveness for infected post-operative wounds, superficial partial-thickness burns, and acute wounds compared to conventional treatments [30]. The main advantages of honey that aid in wound healing are its antimicrobial and anti-inflammatory properties. The primary antimicrobial activity in most honeys is due to the generation of hydrogen peroxide (H_2_O_2_) [31], while the antioxidant capacity of honey is closely linked to the concentration of its phenolic compounds [32]. Honey’s bioactive components, including proteins, acids, phenols, flavonoids, and vitamins, drive its wound healing potential. Its physicochemical properties, such as pH, moisture, sugar content, and enzyme activity, also enhance its effectiveness [33]. Studies also show natural honey aids wound healing by sterilizing infections, promoting tissue growth, enhancing epithelialisation, and reducing scars [34].

**Figure 3:**
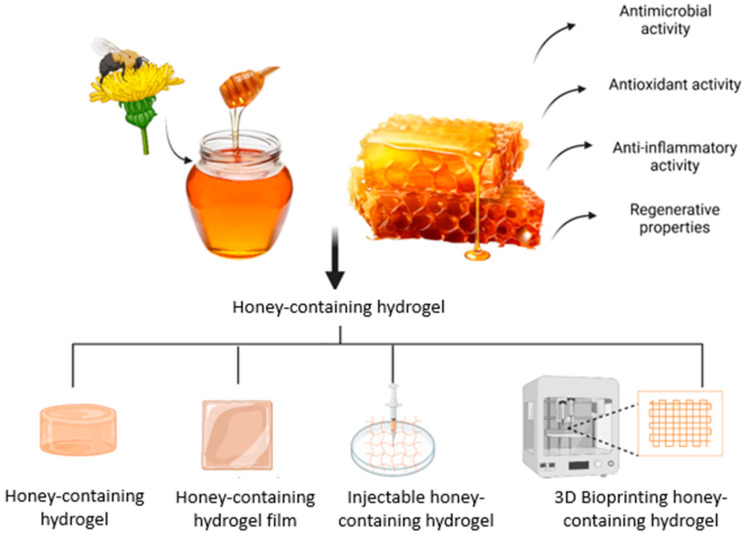
The honey-containing hydrogel that can be fabricated into different forms of hydrogel to aid in wound healing. The idea of this figure is adapted from Rossi et al. (2021) [35].

Incorporating honey into biomaterials shows great promise for wound healing, spurring the development of honey-containing dressings. However, traditional dressings with honey face issues like absorption, poor wound penetration, and short-lived antimicrobial effects. These challenges have led to innovative solutions, such as electrospun fibres and hydrogels, to enhance honey’s effectiveness in wound care [35]. Research indicates that honey-containing hydrogel dressings improve water absorption, reduce bacterial growth, encourage cell proliferation, support epithelialisation, and accelerate wound healing more than other dressings [36,37]. Hence, honey-containing hydrogels are being developed to aim in enhancing wound healing through targeted delivery, sustained antimicrobial effects, improvements in cost, usability, and complication prevention [28]. Due to its potential toxicity, pure honey cannot be directly applied to cells, but embedding it in scaffolds ensures a safe, controlled release of its bioactive compounds [38]. In this review, we present an update on honey’s biological properties on wound healing, the incorporation of honey into hydrogels, and the therapeutic application of honey in in vitro and in vivo studies. Figure 3 shows the honey being incorporated into hydrogels with its properties in enhancing wound healing.

## 2. Data Extraction Management

A literature search was conducted for publications within the past seven years (2018–2024) using platforms such as PubMed, Web of Science (WoS), Scopus, and Google Scholar. The search strategy employed keywords including ‘honey’, ‘hydrogel’, ‘in vitro’, ‘in vivo’, and ‘honey-wound healing mechanisms’. This review focuses on research involving the combination of honey and biomaterials for wound healing. Studies were included if they evaluated the effects of honey-containing biomaterials on the wound healing process, specifically in cells, animals, and human subjects. Exclusion criteria encompassed all the secondary literature and any original articles not written or submitted in English.

## 3. Biological Properties of Honey in Wound Healing

**Figure 4:**
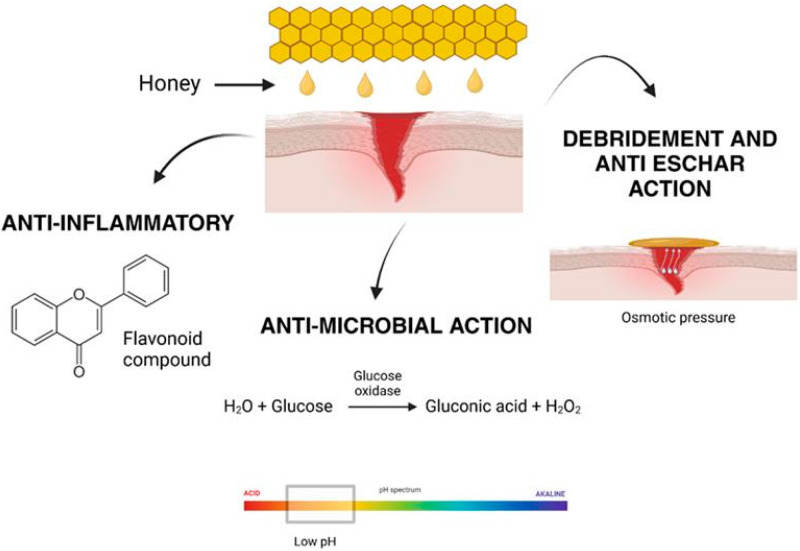
Honey aids in wound healing through its anti-inflammatory properties, antimicrobial effects, and its ability to promote debridement and anti-eschar action by creating osmotic pressure.

Honey has been recognized as a natural remedy for various medical conditions, including wound healing, for centuries [28]. Its effectiveness lies in its unique composition, which supports multiple biological processes essential for tissue repair and regeneration. Honey’s diverse mechanisms of action, including antimicrobial, anti-inflammatory, debridement, and anti-eschar effects, enable it to promote wound healing efficiently and holistically, as shown in Figure 4 [39]. The renewed interest in honey’s therapeutic applications stems from its biocompatibility and its ability to overcome the challenges posed by antibiotic resistance and adverse reactions associated with synthetic drugs.

### 3.1. Antibacterial Effects

**Figure 5:**
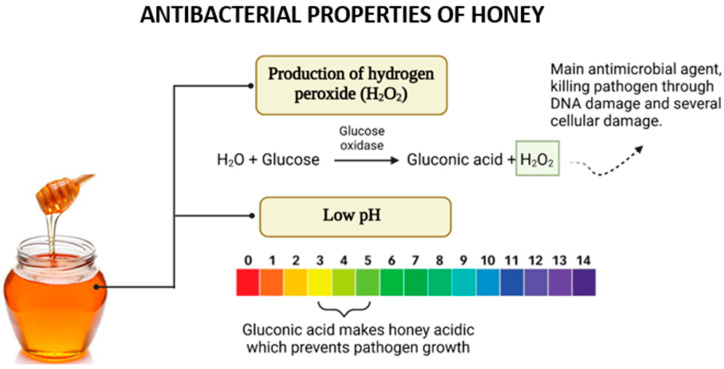
The antibacterial effects of honey arise from its ability to produce hydrogen peroxide (H_2_O_2_) through the action of glucose oxidase and its acidic nature due to gluconic acid. These factors work synergistically to inhibit pathogen growth and enhance antimicrobial activity.

Traditionally, honey was widely used as a natural remedy for infections, especially in wound care. However, its use diminished with the introduction of antibiotics. In recent years, the growing concern over antimicrobial resistance has reignited interest in honey as a natural antibacterial agent. Its effectiveness against microbes is due to a combination of its distinctive physicochemical properties and bioactive compounds, as illustrated in Figure 5.

In most types of honey, hydrogen peroxide (H_2_O_2_) is a critical component contributing to its antimicrobial properties. The production of H_2_O_2_ is facilitated by the enzyme glucose oxidase (GOx), introduced into honey by bees during nectar collection [40]. This enzyme catalyzes the oxidation of glucose to gluconolactone while concurrently reducing molecular oxygen to generate hydrogen peroxide [41].

The role of H_2_O_2_ in the antimicrobial activity of honey, particularly when diluted, has been demonstrated experimentally. Studies revealed that the addition of catalase, an enzyme that decomposes hydrogen peroxide, significantly reduced or eliminated the antimicrobial effect of honey, confirming H_2_O_2_’s central role [28]. Notably, the activity and presence of H_2_O_2_ are more pronounced in diluted honey than in concentrated honey. This is because glucose oxidase regains activity upon dilution, enhancing its access to substrates and thereby increasing hydrogen peroxide production. However, excessive dilution results in a decline in H_2_O_2_ generation due to the reduced substrate concentration available for enzymatic reactions [42]. The acidic pH of honey significantly contributes to its antibacterial activity. This low pH, typically ranging between 3.5 and 4.6, is attributed to the presence of organic acids within honey’s composition, even though these acids constitute only a small proportion (approximately 5%) of its total makeup [43]. The acidic nature of honey provides an effective barrier against microbial contamination. A diverse range of organic acids has been identified in honey, including acetic, aspartic, citric, butyric, fumaric, oxalic, galacturonic, formic, gluconic, glutamic, pyruvic, glutaric, 2-hydroxybutyric, 2-hydroxyglutaric, isocitric, lactic, methylmalonic, malic, malonic, 2-oxopentanoic, propionic, quinic, shikimic, succinic, and tartaric acids [44]. Among these, gluconic acid, produced through the oxidation of glucose in honey, is both the most abundant and the most crucial for its antibacterial activity [45].

The acidic pH of honey has demonstrated effectiveness in suppressing the growth of various microorganisms, including *Escherichia coli*, *Streptococcus pyogenes*, *Salmonella* spp., *Pseudomonas aeruginosa*, and multiple yeasts. Although yeasts are generally more resilient and capable of surviving and reproducing at lower pH levels compared to bacteria, the acidic environment created by honey still poses significant challenges to their proliferation [28]. Thus, the combined effects of hydrogen peroxide and honey’s acidic pH highlight its remarkable efficacy as a natural antimicrobial agent, emphasizing its importance in addressing microbial infections amidst the growing challenge of antibiotic resistance.

### 3.2. Anti-Inflammatory Effects

Honey is also known to demonstrate an anti-inflammatory effect that helps in reducing prolonged inflammatory response. During the inflammatory phase of wound healing, the body will produce an inflammatory response to remove infectious organisms from the body and assist in tissue repair. However, it becomes a problem when prolonged inflammation occurs, which leads to the change in the cell population found at the site of inflammation. The available medications for anti-inflammation mostly are not compatible with wound healing and exhibit cytotoxicity to the tissue, like non-steroidal anti-inflammatory medications (NSAIDs) [46]. Unlike NSAIDS, honey provides a natural alternative that supports tissue repair without causing cytotoxic effects.

The mechanisms of honey in anti-inflammatory effects are complex depending on the phase of wound healing, the microenvironment, and the composition of honey. Notably, honey’s anti-inflammatory properties are largely attributed to its rich abundance of antioxidants, particularly flavonoids and polyphenols [47]. These compounds, especially flavonoids, exhibit anti-inflammatory activity through various molecular mechanisms, including their roles as antioxidants and free-radical scavengers [48]. Free radicals and ROS, generated during normal oxygen metabolism or induced by external factors, pose a constant threat to body cells and tissues [19]. Excessive ROS production can lead to oxidative stress, which is implicated in a wide range of pathological conditions, from inflammatory injuries to cancer. By neutralizing these harmful molecules, the antioxidants in honey help mitigate oxidative damage, thereby alleviating inflammation and protecting cells. This dual role of flavonoids as both anti-inflammatory agents and ROS scavengers underscores honey’s potential as a natural therapeutic agent for managing oxidative stress-related diseases and promoting overall cellular health.

Moreover, the bioactive compounds in honey effectively inhibit the production of pro-inflammatory signalling molecules, such as cytokines and chemokines, by modulating key cellular pathways involved in inflammatory responses. Numerous studies have explored honey’s role in regulating the production of these pro-inflammatory molecules, though the results are inconsistent. For instance, some research has shown that honey can both stimulate [49] and suppress [50] the secretion of interleukin-6 (IL-6), a prominent pro-inflammatory cytokine. These variations in findings may be attributed to factors such as the type and concentration of honey used, as well as the experimental context, including whether the studies were conducted under in vivo or in vitro conditions [51].

The ability of honey to modulate inflammatory pathways, whether by stimulating or suppressing pro-inflammatory molecules like IL-6, plays a crucial role in wound healing. By regulating the inflammatory response, honey helps prevent the persistence of prolonged inflammation, a condition that can hinder the normal progression of the wound healing process and potentially lead to the development of chronic wounds. In addition, honey is also known to help in reducing edema and exudates in the wound area because of inflammation, especially if the inflamed tissue targets the skin [52]. The buildup of fluid and white blood cells in the injured area is due to the release of inflammatory cytokines into the bloodstream [53]. There are a few studies that reported on the anti-inflammatory activities of honey against inflammatory edema in skin using a skin model, the carrageenan-induced paw edema in rodent. They showed that the administration of Gelam honey either orally or intraperitoneally in varying doses effectively reduces inflammation, cytokine production (TNF-α, IL-6, NO, COX-2, PGE2), and pain by inhibiting NF-κB translocation and preventing IκBα degradation [54].

**Figure 6:**
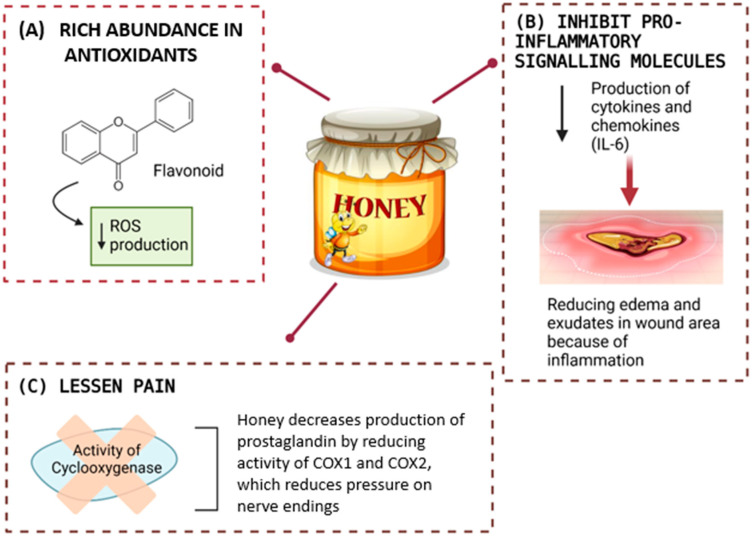
Honey supports healing through its antioxidant properties, which lower reactive oxygen species (ROS) production. It also reduces inflammation by inhibiting pro-inflammatory molecules like cytokines and chemokines (e.g., IL-6) and alleviates pain by suppressing cyclooxygenase (COX1 and COX2) activity, thereby decreasing prostaglandin levels and relieving nerve pressure.

Additionally, this effect of the anti-inflammatory activities of honey also helps to lessen pain by reducing pressure on nerve endings and decreasing the production of prostaglandins during inflammation. This is because honey can intervene in the synthesis of prostaglandin by reducing the activity of cyclooxygenase 1 and 2 (COX1 and COX2) [55]. Thus, honey’s anti-inflammatory properties, driven by its antioxidants, bioactive compounds, and ability to regulate key inflammatory pathways, establish it as an effective natural agent for promoting wound healing. By minimizing prolonged inflammation, suppressing pro-inflammatory cytokine production, reducing edema, and inhibiting prostaglandin synthesis, honey facilitates tissue repair, prevents chronic wound development, and alleviates pain without exhibiting cytotoxic effects. The effects of the anti-inflammatory activities of honey are illustrated in Figure 6. 

### 3.3. Debridement and Anti-Eschar Action

Honey is a hypertonic solution that possesses an osmotic effect. Due to this, it facilitates the process of autolytic debridement. Autolytic debridement is a natural process in which the body’s phagocytic cells and proteolytic enzymes work together to break down necrotic tissues [56]. The high sugar content of honey creates a strong osmotic gradient, drawing fluid from the wound bed and surrounding tissues, which helps cleanse the wound by removing debris and necrotic tissue [57]. This process is essential to retain a moist wound environment to promote healing. Additionally, honey contains protease enzymes that can induce scar tissues to initiate this process [58].

In addition, honey exhibits a preventive effect against eschar formation by blocking the conversion of plasminogen to plasmin, an enzyme that breaks down fibrin within the wound without affecting the collagen matrix. Preventing eschar formation is possible because the collagen matrix is the one necessary in wound healing for tissue re-epithelization [17]. This action is facilitated by honey’s mechanism of inhibiting the production of plasminogen activator inhibitor (PAI) [59]. By limiting the activity of plasmin, honey helps maintain the integrity of the collagen matrix, which is crucial for providing structural support and guiding tissue regeneration.

Clinical trials and studies underscore the effectiveness of honey as a debridement agent for various wound types. In a 15-day study comparing chemical and honey-containing debridement methods in 20 diabetic foot ulcer patients, four groups were assessed: one using a chemical agent and three using different types of honey. The chemical group underwent daily dressing changes, while the honey groups had dressings changed every three days. The findings revealed that chemical debridement was the least effective, whereas 100% debridement was achieved with 100% pure medical-grade Manuka honey [60]. Similarly, a study on spinal trauma patients with stage III/IV pressure ulcers reported 90% complete wound healing within four weeks of Manuka honey application, with wound swabs turning negative after just one week [61].

**Figure 7:**
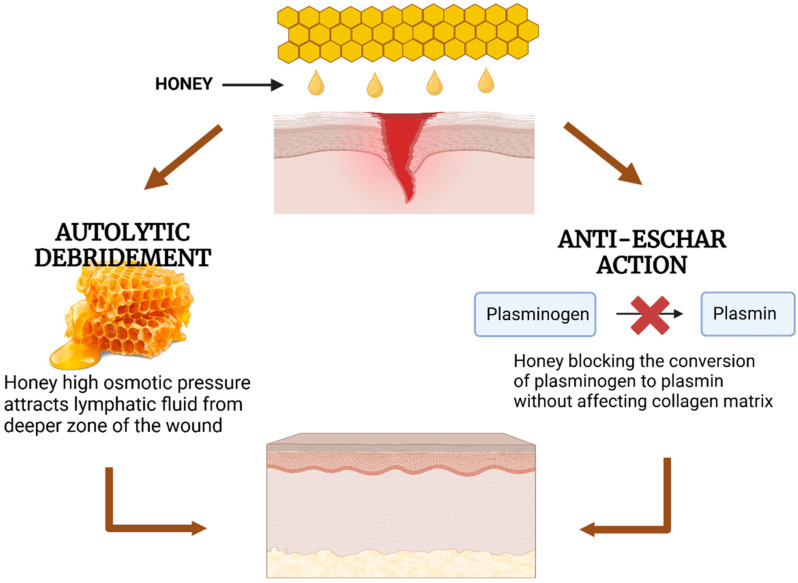
Mechanisms of honey in wound healing through autolytic debridement and anti-eschar action. Honey promotes autolytic debridement by attracting lymphatic fluid due to its high osmotic pressure and prevents eschar formation by inhibiting the conversion of plasminogen to plasmin without degrading the collagen matrix.

Furthermore, its ability to facilitate autolytic debridement and prevent eschar formation shown in Figure 7 ensures optimal wound cleaning and promotes tissue repair. These features position honey as a powerful natural solution for enhancing wound healing and overcoming the limitations of traditional treatments. By harnessing these properties, honey offers a natural and effective approach to advancing wound care and improving patient outcomes.

## 4. Incorporation of Honey into Hydrogels

### 4.1. Hydrogels

Hydrogels are 3D structures with high water absorption and are capable of expanding in aqueous environments due to their hydrophilic components (-NH_2_, -COOH, -OH, -CONH_2_, -CONH, -SO_3_H), giving them flexibility and softness [62]. Typically, its structure involves crosslinked polymer chains, where the cohesion forces enabling crosslinking have a covalent character, along with other interactions such as electrostatic forces, hydrophobic interactions, dipole–dipole interactions, and hydrogen bonds [63]. They are formed through the chemical or physical crosslinking of natural or synthetic polymers and closely resemble living tissue due to their high water content, soft structure, and porous nature. Wichterle and Lim pioneered hydrogels in 1960 with poly-2-hydroxyethyl methacrylate (PHEMA) for eye enucleation and contact lenses. Since then, hydrogels have been widely explored for drug delivery, bioactive compound release, and tissue engineering [64]. Their properties, including swelling rate, stiffness, degradability, and mesh size, can be adjusted by altering factors such as by modifying polymer composition, initiator concentration, and reaction conditions, including time, temperature, and container specifications [65].

Hydrogels are classified by their source, composition, responsiveness to stimuli, crosslinking, properties (e.g., mechanical strength, swelling, porosity, degradability, and adhesivity), configuration, and ionic charge. Hydrogels form through polymer chain crosslinking and are categorized into natural, synthetic, or semi-synthetic hydrogels based on their sources. Natural hydrogels include cellulose, chitosan, collagen, alginate, agarose, hyaluronic acid, gelatin, and fibrin, among others [66]. Natural hydrogels offer biocompatibility, bioactivity, and biodegradability but have weak stability and mechanical strength [67,68]. While generally safe, certain components of natural hydrogels can act as allergens in rare cases, posing potential immunological risks for sensitive individuals [69]. In contrast, synthetic hydrogels are made from human-made polymers synthesized through monomer polymerization, including polyvinyl alcohol (PVA), polyethylene glycol (PEG), polyethylene oxide (PEO), poly-2-hydroxyethyl methacrylate (PHEMA), poly-N-isopropyl acrylamide (PNIPAM), polyacrylic acid (PAA), and polyacrylamide (PAAM). While some, like PAAM, are biocompatible, synthetic hydrogels generally offer greater stability and mechanical strength [66]. Semi-synthetic hydrogels combine natural and synthetic polymers or involve chemically modified natural polymers, such as methacryloyl-modified gelatin (GelMA) and acrylate-modified hyaluronic acid (AcHyA) [70].

Hydrogels can also be classified by their crosslinking method. Chemical hydrogels form permanent junctions via covalent crosslinking and polymerizing end-functionalized macromeres [64]. Chemical crosslinking methods for hydrogel formation encompass various techniques, including chemical reactions, high-energy radiation, free-radical polymerization, and enzyme-based processes [71]. Chemical reactions involve polymer interactions with a crosslinking agent [72]. Both high-energy radiation and free-radical polymerization use free-radical crosslinking, with the former relying on gamma rays or electron beams and the latter on enzyme catalysts or UV excitation [73]. Enzyme-catalyzed crosslinking occurs in polymers modified or infused with enzyme-sensitive molecules [74]. In contrast, physical hydrogels have transient junctions formed through interactions like ionic interaction, hydrogen bonding, and crystallization [63]. Their crosslinking mechanisms also include hydrogen bonding, amphiphilic grafting, block polymer formation, crystallization, and ionic interactions [75]. Hydrogen bonding in hydrogels occurs among molecules with the N-H, O-H, or F-H groups, enabling polymers with these functional groups to form hydrogels. Amphiphilic grafting and block polymer formation rely on the self-assembly of polymers in hydrophobic or hydrophilic solvents due to their amphiphilic nature. Crystallization facilitates hydrogel synthesis by adjusting polymer crystallization temperatures, commonly using freeze–thaw or heating methods. Ionic interactions contribute to crosslinking through the attraction of ionic groups [76].

### 4.2. Fabrication Approach of Hydrogel

#### 4.2.1. Injectable Hydrogel

In recent decades, tissue engineering has developed injectable hydrogels as an innovative method for delivering cells to specific lesion sites, with encapsulated cells sensing their biomechanical environment via focal adhesion [77]. The concept of an injectable hydrogel involves the administration of biomaterials in liquid form, which then undergo in situ gelation at the site of application, forming a solid hydrogel. This process allows for localized and controlled therapeutic effects, making it widely used in biomedical applications. However, not all injectable hydrogels follow this process. Some shear-thinning, self-healing hydrogels are injectable, as they can be administered in gel form [78].

Conventional polymeric hydrogels, designed as prefabricated scaffolds, struggle with treating uneven wounds and encapsulating therapeutic compounds. This has led to growing interest in in situ hydrogels for wound care [79]. Injectable hydrogels are gaining attention in biomedicine for their minimally invasive implantation, which reduces pain and discomfort, accelerates healing, lowers costs, and enables the treatment of difficult-to-reach tissue areas [80]. Injectable hydrogels show great promise for wound healing, as they can be shaped to fit the wound’s form. In instances of deep or irregular wounds, they quickly fill the wound site, aiding recovery [81]. Also, injectable hydrogels have a fluid nature, allowing easy injection and adaptation to wound shapes. They are commonly used to fill deep wounds and offer a more cost-effective solution compared to 3D bioprinting [82]. For example, Masri et al. found that injectable gelatin–PVA (GPVA) hydrogels degraded faster than 3D bioprinted GPVA hydrogels after genipin (GNP) crosslinking [77].

**Figure 8:**
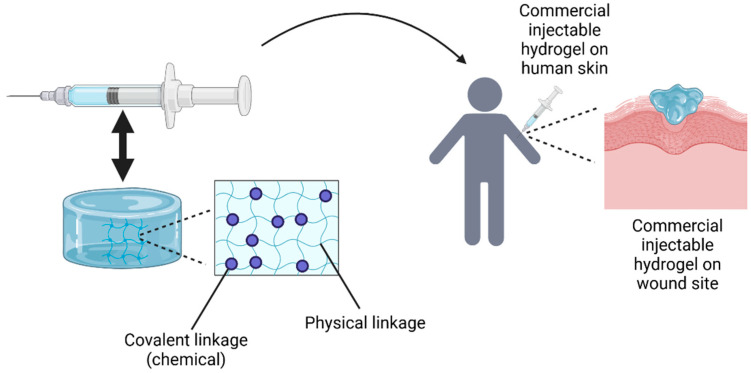
The fabrication of injectable hydrogels involves preparing the hydrogel solution, which is then transferred into a syringe with a nozzle for injection directly onto the cutaneous wound site. The idea of this Figure was adapted from Alonso et al. (2021) [83].

A key factor in designing injectable hydrogels is polymer solution viscosity, which proves beneficial for minimally invasive surgical techniques. However, some hydrogels may cause adverse reactions like inflammation, immune responses, or local and systemic reactions over time [83]. This underscores the significance of biocompatibility and non-toxicity for effective injectable hydrogels. Like other hydrogels, their mechanical properties and durability can be adjusted by modifying monomer ratios, molecular weights, and crosslinking density. To achieve this, conventional crosslinking methods are used to fabricate injectable hydrogels. Currently, injectable hydrogels can be crosslinked through various synthesis mechanisms, creating both physical and chemical linkages that may coexist [84]. Physically crosslinked injectable hydrogels generally have weaker mechanical properties than chemically crosslinked ones. However, chemical crosslinking involves slow gelation kinetics, forming hydrogels in situ upon injection [85]. Figure 8 shows the fabrication of injectable hydrogels and how they can be applied on cutaneous wounds to aid in the wound healing process.

#### 4.2.2. Three-Dimensional Bioprinting Hydrogel

In addition to injectable hydrogels, another fabrication method involves creating 3D hydrogels by using both traditional and advanced 3D bioprinting technologies. Three-Dimensional bioprinting has become a valuable approach in tissue engineering and regenerative medicine, allowing the fabrication of biological substitutes, scaffolds, in vitro drug models, and artificial organs or tissues [86]. Using additive manufacturing, 3D bioprinting layers live cells, biomaterials, and other components to construct 3D bioscaffolds and intricate 3D structures [87]. Since its introduction, 3D printing technology has rapidly advanced in medical and scientific research, now enabling the printing of biological materials including cells, biocompatible materials, and components for functional living tissue fabrication [88]. The procedure involves a systematic process and the automated layering of living and non-living materials using CAD software. Key stages in 3D bioprinting include medical imaging acquisition, 3D bio-modelling, the preparation of inks containing biomaterials (without cells) or cells, actual bioprinting (3D bioprinting) with calibration and slicing, structure maturation, and subsequent physical, chemical, and biological analysis [89].

The integration of biological materials into additive manufacturing, known as biofabrication, increases the complexity of 3D printing and material properties. Biomaterial inks are typically cell-free aqueous formulations with biological factors, mainly composed of polymers or hydrogels. Examples include sacrificial materials like agarose, Pluronic 127, alginate, and gelatin. These materials are printed and subsequently dissolved, leaving behind a structure that does not compromise cell survival [90]. On the other hand, bioinks, composed of cells, biomaterials, and growth factors, are classified as scaffold-based or scaffold-free bioinks. Scaffold-based techniques are widely used in 3D bioprinting, where living cells are encapsulated within biomaterial matrices and bioprinted into pre-designed structures [91]. These substances are called “bioinks” or “cell inks”, as they involve cells combined with a substrate during or applied to a printed surface. They must support cell suspension, facilitate printing, and solidify post-printing to preserve shape accuracy and provide mechanical stability like the native tissue they aim to replace [92].

The effectiveness of bioprinting techniques depends on cell ink properties, including viscosity, cell seeding density, temperature sensitivity, shear stress resistance, and thixotropy/rheopexy behaviour [93]. For example, Hu et al. developed a bioink combining chitosan grafted with polyethylene glycol (PEG), α-cyclodextrin (α-CD), and gelatin for tissue and organ remodelling [94]. Similarly, Zhang et al. created a bioink using silk fibroin and decellularized extracellular matrix (SF-dECM), enriched with bone marrow-derived mesenchymal stem cells, for cartilage tissue engineering scaffolds [95].

### 4.3. Honey and Hydrogel: Physicochemical and Mechanical Properties

Honey-containing hydrogel wound dressings have been widely utilized in the biomedical field. These hydrogels offer numerous benefits and are regarded as optimal wound dressings for enhancing the healing process [46]. Applying honey directly to a wound can be difficult because it tends to spread out over time, leading to patient discomfort. Moreover, while the direct application of pure honey (or other beehive-derived products) might be harmful to cells and tissues, using a suitable carrier like hydrogels or cryogels ensures a controlled, safe, and bioactive release of honey’s therapeutic compounds. Thus, to address this issue, incorporating honey into a hydrogel system proves to be a more practical and effective solution [35]. Studies have demonstrated that honey-infused hydrogels accelerate wound healing by improving physicochemical properties, maintaining a humid environment, and directly aiding tissue regeneration [96].

Numerous methods and fabrication techniques have been explored to optimize the incorporation of honey into hydrogels, ensuring uniform distribution, stability, and the prolonged release of its bioactive compounds. One effective approach to incorporate honey into hydrogels is the cold mechanical method, which stands out among crosslinking techniques due to its ability to operate at room temperature and physiological pH without relying on toxic or difficult-to-remove crosslinking agents [97]. As demonstrated by Abraham et al., the cold mechanical method was used to incorporate honey into a hydrogel. Carbopol 940 and chitosan served as base polymers, initially dissolved in distilled water and hydrated under continuous stirring. Triethanolamine functioned as a buffering agent to neutralize the pH, while methyl paraben acted as a preservative, and propylene glycol served as an emollient. Honey was incorporated during the final stage to ensure uniform dispersion within the gel matrix. This approach enables a controlled and sustained release of honey’s therapeutic properties, enhancing its effectiveness in wound healing applications [98]. Then, another efficient method for incorporating honey into hydrogels is the freeze–thaw technique, a physical crosslinking approach. Physically crosslinked hydrogels have gained preference in recent research due to concerns about the potential toxicity of chemical crosslinking agents, which must be removed before use. However, this removal process can negatively impact the hydrogel’s structural integrity [99]. Koosha et al. incorporated honey into hydrogels using the freeze–thaw method. Chitosan and PVA solutions were prepared separately and blended with honey and allantoin solutions. The mixture was stirred, cast into Petri dishes, and subjected to three freeze–thaw cycles (−20 °C for 18 h, then 25 °C for 6 h) to induce physical crosslinking. Finally, the hydrogel films were left to dry for 24 h to remove the solvent [100].

Apart from the cold mechanical and freeze–thawing fabrication technique, honey can also be incorporated into hydrogels through redox-initiated free-radical polymerization, a type of chemical crosslinking method. As demonstrated by Pinthong et al., Manuka honey was incorporated into the hydrogel using a redox-initiated free-radical polymerization process. It was first mixed into the surfactant solution in vials. These vials, along with a third vial containing methacrylic acid, were thoroughly mixed before being sequentially combined. The polymerization was initiated using a redox pair of Ammonium Persulfate (APS) and N, N, N′, N′-Tetramethyl ethylenediamine (TEMED), ensuring the formation of a porous hydrogel with uniformly distributed Manuka honey [101]. These diverse fabrication techniques highlight the versatility of incorporating honey into hydrogels, each offering unique advantages in terms of biocompatibility, stability, and the controlled release of bioactive compounds. Understanding how these methods influence the physicochemical properties of hydrogels is crucial, as the addition of honey can significantly alter characteristics such as swelling behaviour, mechanical strength, and degradation rates, which are essential for their biomedical applications.

#### 4.3.1. Swelling Ratio Index

One key physicochemical property improved by adding honey to hydrogels is the swelling ratio index, which reflects the hydrogel’s ability to absorb water or fluids, like Dulbecco’s Phosphate-Buffered Saline (DPBS), as well as its crosslinking density and retention capability [102]. The swelling index is shown to increase with higher concentrations of honey, which can be attributed to honey’s hygroscopic nature. This property enhanced the water uptake capacity of the hydrogels [103]. Additionally, a study by Abraham et al. revealed that formulations containing 2% Carbopol with 40% honey and 3.5% chitosan with 40% honey exhibited the highest swelling after 3 h. The study further suggested that the increase in honey concentration led to greater swelling due to the polymer’s viscosity and the porous structure of the hydrogel, which enables rapid solvent uptake by providing a larger surface area [98]. These findings indicate that both the hygroscopic nature of honey and the structural characteristics of the hydrogel contribute to enhanced swelling, aligning with the observations reported in previous studies. Also, Pinthong et al. reported that hydrogels containing Manuka honey (MH) exhibited a controlled swelling behaviour, allowing them to retain their original shape better than hydrogels without MH [101].

However, a study by Koosha et al. found that incorporating honey into PVA–chitosan-based hydrogels significantly reduced the swelling ratio. This effect is attributed to strong hydrogen bonding between honey and PVA, which modifies the polymer network and restricts water absorption. Since honey contains mono- and polysaccharides with hydroxyl groups, it forms hydrogen bonds with PVA chains, limiting the hydrogel’s capacity to swell. In contrast to Abraham et al., where honey increased swelling in chitosan-based hydrogels, the reduction in PVA–chitosan–honey-containing hydrogels is likely due to honey’s solubility effect on the polymer chains. In PVA systems, honey forms hydrogen bonds with both water and the polymer, enhancing solubility and creating a more compact structure that limits expansion [100]. Andriotis et al. observed swelling maxima when Manuka honey was incorporated into hydrogel. However, these extreme conditions were unsuitable due to excessive gelation at high honey concentrations or insufficient gelation at low concentrations [104].

#### 4.3.2. Tensile Strength and Elongation at Break

Another key physicochemical property enhanced by incorporating honey into hydrogels is tensile strength and elongation at break. These properties are vital in tissue engineering to replicate the mechanical strength of natural skin, defining the hydrogel’s performance [99]. Tensile strength and elongation at break are inversely related, and hydrogels with higher tensile strength have lower elongation at break, being less flexible and requiring more force to break [105]. A study by Chopra et al. demonstrated that incorporating raw honey into hydrogel films influenced tensile strength, ranging from 4.74 ± 0.83 to 38.36 ± 5.39 N, and elongation at break, ranging from 30.58 ± 3.64 to 33.51 ± 2.47 mm. However, the study attributes the mechanical robustness primarily to the strong interactions between chitosan and PVA [106]. Simultaneously, the ultimate tensile strength of chitosan-based hydrogels decreased with an increasing honey concentration, while the highest elongation at break was observed at higher honey concentrations [99]. This indicates enhanced strain and flexibility due to the plasticizing effect of honey in the hydrogels [36]. However, Koosha et al. observed opposite results, where hydrogel films containing honey exhibited the lowest tensile strength with the highest elongation at break [100].

#### 4.3.3. Water Vapour Transmission Rate (WVTR)

The water vapour transmission rate (WVTR) is an important property of honey-containing hydrogels. It measures how fast water vapour passes through the hydrogel, which is crucial for applications like wound dressings. Proper moisture management helps healing and prevents fluid buildup. The right moisture permeability maintains a moist environment for wound recovery and stops excessive exudate loss, which could cause dehydration. An ideal dressing material should have a WVTR of 2000–2500 g/m^2^/day [107]. A study found that the WVTR of the hydrogel films ranged from 1650.50 ± 35.86 to 2698.65 ± 76.29 g/m^2^/day when honey was being added into the formulations [106]. A study by Saberian et al. discovered that the WVTR in aloe vera–chitosan–honey hydrogel decreased significantly to 380.4 ± 21.50 g/m^2^/day, likely due to the high viscosity of honey in the formulation. However, despite this reduction, the moisture flow rate remained within an acceptable range [108].

#### 4.3.4. In Vitro Biodegradation Test

Moreover, in vitro biodegradation tests are another critical physicochemical property of honey-containing hydrogels. These tests show how the hydrogel degrades under simulated physiological conditions, helping predict its longevity and effectiveness in vivo [109]. The hydrogels’ moist nature, along with their hydrophilic and biodegradable properties, helps reduce pain from frequent dressing changes and lowers the risk of additional wound damage [110]. Mukhopadhyay et al. found that the degradation rate of honey–sodium alginate-based hydrogel (HSAG) increased with time and higher honey concentrations. This might be due to the swelling study suggesting that higher honey concentrations favoured better water uptake, leading to the formation of an aqueous environment that facilitated degradation [103]. Similarly, Koosha et al. observed that honey-containing samples showed a higher mass loss and a faster in vitro biodegradation rate at 37 °C in Phosphate-Buffered Saline (PBS). This can be attributed to the presence of low-molecular-weight saccharides in honey, which dissolve more easily in water [100]. Shamloo et al. observed comparable results, where hydrogels with higher honey concentrations (10% and 20% *v*/*v*) demonstrated faster degradation compared to those with lower concentration (5% or 0% *v*/*v*) honey-containing hydrogels. The accelerated degradation was mainly attributed to honey’s high solubility in water [99]. The weight loss of polyvinylpyrrolidone (PVP) combined with honey was reported to range from 15 to 20%. This weight loss indicated that honey began to decompose, disrupting the coupling between PVP and honey, while the crystal water was released during the process [111].

#### 4.3.5. Contact Angle and Viscosity

Contact angle (CA) measurement is another important property to evaluate when honey is incorporated into hydrogels. This test determines the hydrophilicity or hydrophobicity of the hydrogel surface, which affects its wettability, surface energy, and cell adhesion [112]. It was found that CA measurements indicated that higher honey concentrations decreased the hydrophilicity of the hydrogel surface, as shown by increased CA values. This might be because incorporating higher concentrations of honey disrupted the balance of free water on the surface, and the honey’s hygroscopic nature alone was insufficient to maintain the surface’s hydrophilicity [103]. This aligns with the findings of Sebarian et al., who reported that groups containing 20% *v*/*v* honey exhibited reduced hydrophilicity, with a CA of 87.3° [108]. Viscosity is another key parameter to consider when incorporating honey into formulations. It measures a fluid’s resistance to shear stress or the friction between its layers as they flow or slide past each other [113]. The viscosity of hydrogels affects the penetration of active ingredients through the skin. A study by Salva et al. showed that incorporating honey into the chitosan–HA-based hydrogel formulation reduced its viscosity [96]. El Kased et al. reported similar findings, demonstrating that the viscosity of hydrogels decreases with higher honey concentrations in honey–chitosan or honey–Carbopol 934 formulations [97]. This reduction in viscosity allows the hydrogel to flow more easily from containers and improves honey diffusion within the hydrogel network, promoting a smoother application [96].

#### 4.3.6. Porosity

In addition, porosity is also a crucial factor in developing honey-containing hydrogels, as diffusion gradients mainly influence transport properties. Pore size and distribution are key design considerations, significantly affecting hydrogel performance in applications like diagnostics, therapeutics, drug delivery, and cell encapsulation [114]. A study by Pinthong et al. revealed that the incorporation of 1% Manuka honey (MH) led to a reduction in porosity, while the addition of 10% MH increased porosity within the hydrogel matrix. The authors suggested that the addition of 10% MH led to an increase in porosity by creating more pores within the hydrogel structure. This was due to two factors: the alteration of the system’s viscosity and the slightly acidic nature of MH, which affected both the size and duration of gas bubbles formed during the hydrogel’s preparation [101]. However, a study by Saberian et al. showed the opposite result, revealing that the alginate–chitosan–honey hydrogel with 20% *v*/*v* honey had lower porosity compared to other groups and exhibited higher density. This was attributed to the presence of honey in the hydrogel’s formulation [108].

#### 4.3.7. Fourier Transform Infrared Spectroscopy (FTIR)

Next, chemical characterization is essential when incorporating honey into hydrogels. Techniques like Fourier Transform Infrared Spectroscopy (FTIR), Scanning Electron Microscopy (SEM), and Differential Scanning Calorimetry (DSC) are widely used to analyze their properties [45]. Chopra et al. found that the FTIR spectroscopy of honey showed carbohydrate, water, and organic acid vibrations at 3700 cm^−1^ and 3000 cm^−1^. A peak at 2850 cm^−1^ in PVA spectra after interacting with honey indicated honey’s role in crosslinking the hydrogel [106]. Abraham et al. observed similar peaks in their FTIR analysis of honey, including an O–H stretch at 3415.7 cm^−1^ and a C–H stretch at 2891.10 cm^−1^. FTIR analysis of honey with Carbopol 940 showed peaks at 3369.41 cm^−1^ (O–H stretch) and 2873.74 cm^−1^ (C–H stretch). In contrast, honey with chitosan showed prominent peaks at 3618.21 cm^−1^ (O–H stretch) and 3498.63 cm^−1^ (N–H stretch) [98]. These studies verified the compatibility of honey with the polymers, as no physical interactions were detected between them. Conversely, the FTIR spectra of honey-containing formulations showed characteristic peaks for glucose and fructose, including C–H and C=O stretching at 2910 cm^−1^ and 1650 cm^−1^, respectively, and a C–O stretch at 1054 cm^−1^. A broad band at 3300 cm^−1^ indicated O–H stretching vibrations from sugars bonded with water [96].

However, Mukhopadhyay et al. observed FTIR peaks that differed from those in other studies. Their FTIR spectroscopy identified absorption peaks at 778, 818, 1076, 1261, 1417, and 1634 cm^−1^, which were attributed to the presence of glucose, sucrose, and fructose in honey [103]. On the other hand, FTIR analysis by Hu et al. revealed that the peaks of pure PVP at 3393 and 3285 cm^−1^ shifted to 3372 and 3264 cm^−1^ upon compositing with pure honey, which displayed a peak at 3272 cm^−1^. This shift indicates the formation of hydrogen bonds between the OH groups of honey and the C=O groups in the pyrrolidone ring of PVP [111].

#### 4.3.8. Scanning Electron Microscopy (SEM)

In addition to FTIR, SEM is a key technique for chemical characterization, widely used to visualize materials at the microscale and provide detailed insights into the surface morphology and structure of hydrogels and other materials [115]. SEM results showed that the average pore size of the hydrogels increased with higher honey concentrations. However, excessive honey in the polymer solution disrupted the formation of a uniform sponge-like structure [99]. However, Pinthong et al. reported contrasting findings, as SEM micrographs revealed a reduction in average pore size upon the addition of Manuka honey to the hydrogel [101]. For instance, SEM analysis from the Scalzone et al. study revealed that the gellan gum–methacrylic anhydride–Manuka honey samples had 73% of pores smaller than 150 µm, with 44% in the 100–150 µm range and 29% under 100 µm [116].

#### 4.3.9. X-Ray Diffraction (XRD) and Differential Scanning Calorimetry (DSC)

XRD is another effective non-destructive method for characterizing hydrogels. Using X-ray scattering, it identifies crystalline materials, with diffraction patterns providing insights into the atomic arrangements and crystallinity of the hydrogel structure [117]. Mukhopadhyay et al. observed intensified XRD peaks at 2θ angles (17.71°, 30.26°, and 33.78°) after incorporating honey, along with additional peaks at 39.51°, 18.93°, and 17.22°. These findings suggest that honey interacts with sodium alginate through inter-hydrogen bonding, influencing the hydrogel’s crystalline structure. While hydrogels naturally exhibit both crystalline and amorphous properties, the results indicate that honey modifies the structural arrangement rather than increasing crystallinity or amorphousness. This interaction suggests that honey becomes physically entrapped within the hydrogel network, altering its structural organization and properties [103]. Then, DSC is an effective method for analyzing the thermal properties of hydrogels. By calibrating temperature and heat flow with standard materials like indium, DSC detects phase transitions, such as melting or crystallization, and assesses the thermal stability of the hydrogel structure [118]. For instance, a study by Koosha et al. showed that the thermal behaviour of the chitosan/PVA film with honey, as revealed by DSC thermograms, was significantly altered. Three endothermic peaks were observed at 168 °C, 232 °C, and 258 °C. The peak at 232 °C likely corresponds to the melting of the PVA crystals, with a shift to higher temperatures due to strong hydrogen bonding with honey. The peak at 168 °C may be related to the melting of sugar crystals like glucose, fructose, and maltose, while the peak at 258 °C could be due to the melting of other honey components such as minerals, carbohydrates, or enzymes [100].

#### 4.3.10. Honey-Incorporated Hydrogels Using 3D Bioprinting

Additionally, 3D bioprinting has emerged as an innovative method for creating honey-containing hydrogels, especially in tissue engineering. These hydrogels, which mimic natural extracellular matrices, provide an ideal environment for cell encapsulation. Their highly hydrated and mechanically supportive nature enhances their potential for applications in creating functional tissues and organs [119]. A study by Scalzone et al. demonstrated that 5% *w*/*v* Manuka honey (MH) enhanced the printability of gellan gum–methacrylic anhydride (GGMA, 2% *w*/*v*) using a 3D bioprinting approach. When combined with cells to create bioinks, MH improved 3D bioprinting performance by producing more stable and viscous filaments, resulting in better resolution. The GGMA-MH blend had a spreading ratio of 3.5 ± 0.1, with a more uniform diameter distribution. Additionally, MH incorporation allowed for the extrusion of longer fibres compared to bare GGMA, which formed shorter, droplet-shaped filaments [116]. Another study by Andriotis et al. explored the development of bioactive patches using 3D bioprinting with Manuka honey incorporated into pectin-based formulations. The results showed minimal shape deviation from the theoretical dimensions of the patches [104].

Moreover, cell viability is a key factor in 3D bioprinting, influenced by bioink viscosity, crosslinking techniques, and printing conditions [120]. Scalzone et al. studied hydrogels with Manuka honey (MH) in a 3D bioprinting approach using a human TERT-immortalized bone marrow stromal cell line differentiated into chondrocytes (MSCs-C). They observed higher cell death in GGMA-MH samples on days 1 (9.5 ± 3.5%) and 3 (18 ± 6.0%) compared to GGMA alone (2.0 ± 0.5% and 1.8 ± 0.4%). However, metabolic activity remained stable over 7 days. Cells in GGMA-MH constructs also tended to cluster by day 7, likely due to MH’s viscosity and non-uniform pore structure. These results suggest that honey-containing bioinks can support cell viability in 3D bioprinting, but further refinement is needed to enhance uniformity and cell distribution [116]. Also, Brites et al. explored the use of 40% *w*/*w* Manuka honey (MH) in a gelatin-based hydrogel patch for wound healing using 3D bioprinting. Cell viability was evaluated using MTT assay in human dermal fibroblasts (HDFs) and human epidermal keratinocytes (HEKs). The gel patch maintained HDF viability above 77%, while the 3MH-gel (three layers of MH-gel ink) caused a ~50% reduction. The 2MH-gel (layers 1 and 3 of MH-gel ink and layer 2 of gel ink), was the most favourable, increasing metabolic activity by ~20% compared to the control. In HEK cultures, the gel patch sustained viability above 75%, whereas the 3MH-gel reduced it by ~50%. The 2MH-gel initially showed an inductive effect at 6 h but stabilized at ~70% viability after 24 h. The reduced viability in 3MH-gel was likely due to the acidic pH of MH and osmolarity imbalances. However, toxicity was only observed at the highest MH concentration (40%), while 2MH-gel (~27% MH) remained non-cytotoxic and even promoted slight viability enhancement in HDF cells. These results suggest that lower concentrations of MH (~27%) in 3D bioprinted hydrogels are non-cytotoxic and may promote cell activity, while higher concentrations (40%) reduce cell viability [121].

Hence, studies to date have concentrated on developing suitable hydrogel matrix compositions and honey that exhibit beneficial properties, particularly in promoting wound healing. The field of honey-containing hydrogels for wound healing therapies is growing rapidly. However, the progress of these hydrogels is often limited by the scarcity of clinical trials that could establish definitive evidence of their safety and therapeutic efficacy. The successful bio fabrications of honey-containing hydrogel are listed in Table 1.

## 5. Therapeutic Applications of Honey: In Vitro and In Vivo Studies

Honey, well known for its ancient medicinal properties, has received significant attention in modern therapeutic research, particularly for its application in wound healing. Experimental studies play a crucial role in validating the wound healing properties of honey, demonstrating its effectiveness through various mechanisms such as antimicrobial, anti-inflammatory and immunomodulatory effects. In vitro studies provide insights into specific cellular mechanisms, demonstrating honey’s capabilities in promoting healing. Similarly, in vivo studies highlight its practical applications, such as accelerating wound closure, reducing infections and improving overall recovery. Incorporating honey into hydrogels has shown promising results, reducing pro-inflammatory cytokines and fostering processes like angiogenesis, re-epithelization, and granulation tissue formation [122].

### 5.1. In Vitro Studies

In vitro studies have extensively examined the diverse biological properties of honey, with a strong focus on its antibacterial and wound healing potential. Conducted in controlled laboratory settings, these studies evaluate the biological activities of different honey varieties, shedding light on their effectiveness against microbial infections through methods like agar-well diffusion assays. The distinctive composition of honey—comprising natural sugars, phenolic compounds, and enzymes—underpins its antimicrobial efficacy, positioning it as a valuable focus of research in natural medicine and clinical applications. Chopra et al. conducted an in vitro investigation which determined that a hydrogel containing honey demonstrated notable antimicrobial effectiveness, exhibiting a substantial bacteriostatic effect with an inhibition zone diameter measuring 5.01 ± 0.32 mm. This outcome was attributed to the acidic pH and positively charged properties of honey, which interacted with the negatively charged cell membranes of microorganisms, thereby impeding their activity and potentially leading to cell death [106]. Meanwhile, another study by Gopal et al. utilized cellulose hydrogels, incorporating three varieties of honeys from mainland Southeast Asia, sourced from stingless bees, giant bees, and Asian bees, respectively, demonstrating remarkable antibacterial properties. The study observed varying effects of different honey types on different microorganisms; for instance, Kelulut honey hydrogels exhibited slightly larger inhibition zones against E. coli compared to Tualang honey hydrogels, whereas Tualang honey hydrogels displayed greater inhibition zones against S. aureus compared to Kelulut honey hydrogels. Nevertheless, both honey-containing hydrogels recorded optimal cell viability. These findings underscore the potential of honey-containing hydrogels as a dependable alternative for treating wound infections, facilitating cell proliferation while concurrently averting infections in wound areas [37].

A study conducted by Lo et al. revealed that aphthous stomatitis, commonly known as mouth ulcers, treated with a cellulose hydrogel infused with stingless bee honey exhibited a notable cell viability rate of 90%. The honey was released into the extracellular matrix, swiftly closing the wound gap. Furthermore, the honey-containing hydrogel patches demonstrated antibacterial activity by inhibiting the growth of *E. coli* within the initial 2 h, followed by the suppression of *S. aureus* in the subsequent 2 h. These findings highlight the therapeutic potential of honey-containing hydrogels in promoting wound healing by effectively combating bacterial infections [123]. The use of Manuka honey in nanofibrous wound dressings in a study by Zekry et al. exhibited remarkable antibacterial efficacy against *both S. Aureus* and *E. coli*. Notably, scaffolds infused with bee venom (BV) exhibited enhanced efficacy against *S. aureus* compared to those without BV. Cytotoxicity assessments on L929 fibroblast cells further confirmed the biocompatibility of all scaffolds, as they maintained 100% cell viability without any toxic effects [124]. In other applications, Brites et al. developed an innovative 3D patch for regenerative wound treatment by combining a gelatin-based hydrogel with the renowned natural antibacterial agent, Manuka honey (MH). These 3D Manuka–gelatin patches exhibited potent antibacterial properties while significantly enhancing the proliferation of human dermal fibroblasts and human epidermal keratinocytes. The incorporation of honey not only improved the biological responses but also facilitated the ease of 3D printing, making these patches highly promising candidates for advanced wound care applications [121].

Samraj et al. fabricated a nanofibrous membrane using a combination of honey, curcumin, and gelatin. Through in vitro wound scratch assays, they determined that these honey-containing hydrogels were not harmful to fibroblast cells and did not disrupt the natural wound healing process. The in vitro wound scratch assay simulates tissue disruption in real wounds by creating a scratch in a cell monolayer, enabling the study of cell migration and wound healing in a controlled environment. Although it does not fully capture the complexity of real wounds, including inflammation and 3D tissue interactions, it serves as a straightforward and reliable model for initial screening. Additionally, research indicates that the hydrogen peroxide (H_2_O_2_) found in honey plays a pivotal role in promoting wound regeneration by elevating extracellular calcium levels [125]. The release of H_2_O_2_ from honey at the wound site influences multiple wound healing pathways, exerting intricate effects on various aspects of cellular behaviour. These include promoting cell proliferation, modulating signalling pathways, enhancing metabolic activity, and facilitating cell migration, all of which are essential for effective wound repair [28]. Another study by Bonifacio et al. highlights the therapeutic potential of honey in tissue engineering, particularly for cartilage repair. The researchers developed composite scaffolds, incorporating gellan gum, Manuka honey (MH), and three different inorganic clays. These scaffolds demonstrated significant antibacterial properties, effectively protecting human mesenchymal stem cells (hMSCs) co-cultured with staphylococcal strains. The scaffolds supported the growth and chondrogenic differentiation of hMSCs in vitro, underscoring honey’s dual role in promoting stem cell differentiation and enhancing antibacterial responses, making it a promising component in regenerative medicine [126]. A study by Mukhopadhyay et al. demonstrated the development of a dual-crosslinked sodium alginate honey–sodium alginate-containing (HSAG) hydrogel, showcasing the remarkable impact of honey incorporation on the material’s properties and biological performance. The results revealed that increasing honey concentration significantly enhanced the hydrogel’s swelling index, which is critical for creating a hydrated microenvironment conducive to cell activity. This modification facilitated improved cell attachment, proliferation, and growth, highlighting honey’s potential as a bioactive additive in hydrogel systems for tissue engineering and regenerative medicine applications [103].

Beyond hydrogels, numerous studies have explored innovative approaches to harness and maximize the therapeutic potential of honey. These include integrating honey into other biomaterials such as polycaprolactone, methylcellulose, and similar compounds to accelerate the wound healing process. In some cases, electrospinning techniques have been employed to fabricate honey-containing healing mats, further enhancing their efficacy. A study by Schuhladen et al. demonstrated that incorporating Manuka honey as a crosslinking agent with bioactive glass produces dual therapeutic effects, significantly enhancing wound healing properties. This was evident in experiments with human dermal fibroblasts and HaCaT cells, particularly through improved wound migration rates and increased cell viability [127]. Gaydhane et al. investigated nanofibrous mats incorporated with honey and curcumin, which demonstrated a combination of anti-inflammatory, enhanced antioxidant, and moderate antibacterial properties. The heightened antioxidant activity was particularly noteworthy, as it promotes cellular proliferation, a critical factor in accelerating the wound healing process [128].

The therapeutic applications of honey in vitro, emphasizing the critical role of biomaterial structure in its effectiveness, were also studied by Hixon et al. (2019), where Manuka honey was incorporated into different forms of biomaterials; cryogels, hydrogels, and electrospun scaffolds to investigate the impact of scaffold geometry on bacterial clearance, adhesion, and cellular interactions. The findings revealed that the nanoporous fibrous structure of electrospun scaffolds facilitated a faster release of MH, resulting in greater bacterial clearance and reduced bacterial adhesion. In contrast, the more three-dimensional structures of hydrogels and cryogels supported extended MH release, promoting sustained cellular adhesion and prolonged therapeutic effects. This study underscores the importance of tailoring biomaterial architectures to optimize honey’s therapeutic potential for both short- and long-term applications in wound healing and tissue regeneration [129].

### 5.2. In Vivo Studies

In vivo investigations delve into the intricate mechanisms by which honey interacts with biological systems, shedding light on its efficacy in wound healing, inflammation modulation, and even disease prevention. In addition to the in vitro analyses conducted by Zekry et al., the same honey-loaded scaffolds were further evaluated in vivo using a dorsal wound model in adult female Sprague Dawley rats. The study demonstrated a significant increase in wound closure at days 3, 5, and 10 across all treatment groups compared to PVA and no-treatment controls (*p* < 0.0001). Notably, all treatment groups, except for the control group, achieved approximately 98% wound closure by day 10 [124]. Iryani et al. conducted an in vivo study on dermal wound healing, revealing that the integration of honey into virgin coconut oil hydrogel significantly hastened wound closure, achieving an impressive 98 ± 1% closure within just one week. Furthermore, the treatment resulted in complete neo-epidermal formation at the wound site. This accelerated healing process was attributed to honey’s ability to stimulate angiogenesis and promote the growth of fibroblasts, facilitating tissue regeneration at the wound margins [130]. In another study, Samraj et al. applied a membrane made of curcumin and honey topically to wounds in Wistar rats, observing enhanced wound healing compared to untreated groups. This underscores honey’s efficacy in promoting healing, attributed to its anti-inflammatory properties which aid in tissue debridement, scar inhibition, regeneration stimulation, and the maintenance of a moist wound environment [125].

Honey’s potential in wound healing applications has inspired researchers to develop innovative materials that harness its properties to accelerate the healing process. Among these, nanocomposite hydrogels have gained attention for their ability to combine the natural benefits of honey with advanced material functionalities. Noori et al. developed a novel responsive nanocomposite hydrogel utilizing poly(vinyl alcohol)/chitosan/honey/clay as an advanced wound dressing. Their in vivo wound healing assessment revealed that the honey-containing hydrogel nanocomposite dressing (PCMH) exhibited superior wound healing efficacy compared to PCM and control groups. Specifically, three days post-operation, PCMH demonstrated the highest calculated reduction in wound size at 44.24% compared to 43.18% for the PCM group and 39.62% for the control group. By six days post-operation, the reduction in wound size reached a peak at 72.60% for PCMH, while the PCM and control groups showed reductions of 58.38% and 55.23%, respectively. These results highlight the enhanced wound healing ability of the PCMH nanocomposite hydrogel compared to both the PCM group and the control group treated with sterile gas [36].

Honey’s therapeutic potential continues to gain attention in wound care, particularly in the development of advanced hydrogels. A study by El-Kased et al. highlights the therapeutic potential of combining honey with chitosan, a material renowned for its biocompatibility and wound healing properties, to create an effective hydrogel. Their research demonstrated the honey–chitosan hydrogel’s efficacy in promoting wound healing in burn-induced injuries in mice. The 75% honey–chitosan hydrogel not only exhibited the highest burn healing rate compared to a commercial product but also created a moist environment essential for wound repair. Its strong antibacterial properties ensured sterile conditions within 7–10 days of treatment. Histopathological analyses further revealed effective epidermal regeneration and the formation of new blood capillaries, highlighting honey’s significant role in tissue repair and reducing inflammation. This underscores its potential as a safe and effective natural topical treatment [97].

The combination of honey with alginate has shown great promise in advancing wound care treatments. Mukhopadhyay et al. explored this synergy through honey–alginate gels (HSAGs), demonstrating their potential to significantly enhance wound healing. Honey–alginate gels (HSAGs), in a study by Mukhopadhyay et al., demonstrated significant in vivo potential for wound healing, with 4% honey concentration proving optimal. These hydrogels enhanced biosorption, acted as effective wound barriers, and accelerated healing. By the 12th day, 4% HSAG-treated wounds achieved 94.56% contraction (*p* < 0.0001), outperforming negative controls (77%) and alginate-only gels (79%). Early-stage improvements were evident, with rapid healing observed by the 8th day, and treated wounds showed hair regrowth with minimal scarring. The synergistic effect of honey’s bioactive components with alginate highlights its efficacy in promoting wound closure and improving overall healing outcomes [103].

Another study by Rafati et al. explored the therapeutic applications of honey in vivo by utilizing honey-containing hydrogel wound dressings. The findings revealed significant improvements in wound area reduction and healing rates. The honey-containing hydrogels outperformed even advanced wound dressings like Chitopad (used as a control), demonstrating faster healing rates. This enhanced efficacy can be attributed to their ability to create and sustain a continuously moist environment on the surface of both infected and non-infected wounds, which is crucial for optimal healing. Furthermore, the natural antibiotic properties of honey contributed to accelerated wound recovery, highlighting its potential as a key component in advanced wound care products [131]. Furthermore, research conducted by Shamloo et al. investigated the efficacy of various concentrations of chicory-origin honey in expediting wound healing. Optimal concentrations of 10% and 20% were selected for in vivo experimentation on full-thickness wounds in Wistar rats. Results indicated a significant acceleration in wound closure, particularly with the 20% honey concentration, which achieved nearly 50% wound closure within the initial 4 days and reached 95% wound contraction by day 12. This expedited healing process may be attributed to honey’s capacity to stimulate angiogenesis and fibroblast growth, thus facilitating accelerated wound recovery [99].

Table 2 provides a summary of studies that explore the incorporation of honey into biomaterials and various types of scaffolds, highlighting their diverse therapeutic applications, including wound healing.

## 6. Challenges and Limitations

Wound healing is a complex process involving the restoration of damaged tissues and cellular structures. Hydrogel wound dressings have emerged as a promising solution due to their rapid healing capabilities, ability to maintain a moist environment, biodegradability, and protection against bacterial infections. The incorporation of honey into biomaterials, particularly hydrogels, presents significant potential for wound healing applications. For optimal therapeutic outcomes, honey-containing hydrogels must be precisely delivered to the targeted wounded tissue. This review highlights the mechanisms by which honey promotes wound healing, emphasizing its antimicrobial, anti-inflammatory, and anti-eschar effects. It discusses the numerous types of honey used in hydrogel fabrication, explores their therapeutic applications through in vitro and in vivo studies, and summarizes the main outcomes related to cellular and tissue healing processes documented in the literature. However, their application also presents challenges. One major concern is the risk of allergic reactions, as honey contains pollen (especially Compositae pollen) and bee-derived proteins, such as royal jelly, which may trigger hypersensitivity. This necessitates careful patient selection and patch testing before application. Additionally, honey’s composition varies by floral and geographical origin, making standardization challenging and potentially affecting its efficacy. Further research is needed to refine the purification methods that minimize allergens while preserving its benefits. Various biomaterials and hydrogels incorporated with different types of honey have been reported to significantly enhance the regeneration of damaged skin tissues. However, the application of honey-containing hydrogels through injectable and 3D bioprinting techniques remains underexplored, highlighting an important avenue for future research.

## 7. Future Directions

Honey-containing hydrogels offer a promising solution to wound healing challenges, with significant potential in skin tissue engineering and regenerative medicine. Their versatility allows for processing into various structures and shapes using diverse fabrication methods, enhancing their tissue regeneration capabilities. Researchers have the opportunity to investigate various types of honey, each containing distinct active compounds that may offer enhanced wound healing properties for therapeutic use. Future research could focus on developing different honey-containing biomaterials or scaffolds by using an injectable or 3D bioprinting fabrication approach to advance wound healing treatments and tissue regeneration so that it could be aimed to regulate and extend the release of honey from templates to minimize cytotoxicity and sustain its beneficial effects at the target site. At the same time, we recommend the use of biodegradable and eco-friendly materials to address pollution concerns and minimize waste production.

## 8. Conclusions

Experimental evidence from both in vitro and in vivo studies indicates that various types of honey effectively eliminate bacteria, reduce inflammation, and accelerate wound healing. Additionally, honey-containing hydrogels demonstrate outstanding and improved physicochemical properties, including maintaining an optimal swelling ratio index, water vapour transmission rate (WVTR), contact angle, tensile strength, elongation at break, in vitro biodegradation rate, viscosity, and porosity. Thus, honey proves to be a significant addition to tissue engineering scaffolds, especially hydrogels, aiding wound healing, resolving inflammation, and enhancing tissue integration.

## Figures and Tables

**Table 1:** Physicochemical and chemical properties of honey-containing hydrogel.

Honey Types	Fabrication Approach	Physicochemical Properties	Chemical Properties	References
Raw honey (Punjab, India)	Hydrogel films (solvent-casting method)	WVTR ranged from 1650.50 ± 35.86 to 2698.65 ± 76.29 g/m^2^/day Tensile strength ranged from 4.74 ± 0.83 to 38.36 ± 5.39 N Elongation at break ranged from 30.58 ± 3.64 to 33.51 ± 2.47 mm	FTIR peaks at 2850 cm^−1^, 3700 cm^−1^, and 3000 cm^−1^ DSC thermograms (thermal behaviour) revealed 3 endothermic peaks at 103.06 °C, 27.94 °C, and 143.86 °C	Chopra et al. (2022) [106]
Chicory honey	Hydrogel (freeze–thaw method)	Tensile strength ranged between 1.51 ± 0.16, 1.30 ± 0.10, and 1.10 ± 0.12 for 5%, 10%, and 20% *v*/*v* honey, respectively Elongation at break was observed at 46.8 ± 6.2% and 44.9 ± 4.8% for 10% and 20% *v*/*v* honey–chitosan hydrogels, respectively	SEM revealed average pore size of 38 ± 7 µm, 40 ± 9 µm, and 45 ± 10 µm for hydrogel with 5%, 10%, and 20% *v*/*v* of honey, respectively	Shamloo et al. (2021) [99]
Raw honey (Mumbai, India)	Dual crosslinked 3D hydrogel	Weight loss ranged between 87.36%, 95.93%, 98.36%, 98.41%, and 99.29% for 4%, 0%, 2%, 6%, and 10% wt.% honey, respectively Contact angle ranged from 39.73 ± 0.7° to 46.39 ± 0.5° for 2% and 4% wt. honey, respectively	FTIR peaks at 778 cm^−1^, 818 cm^−1^, 1076 cm^−1^, 1261 cm^−1^, 1417 cm^−1^, and 1634 cm^−1^ XRD shows 2θ = 17.71°, 30.26°, and 33.78° with honey incorporation (4%, 6%, and 10%, respectively)	Mukhopadhyay et al. (2020) [103]
Malabar honey	Hydrogel (cold mechanical method)	Swelling index ranged from 65 to 70% after 3 h for Carbopol–Malabar honey and chitosan–Malabar honey-based hydrogel, respectively	FTIR peaks at 3369.41 cm^−1^, 1056.92 cm^−1^, 1423.18 cm^−1^, 2873.74 cm, 3618.21 cm^−1^, 1058.90 cm^−1^, and 3498.63 cm^−1^	Abraham et al. (2022) [98]
Raw honey (Balparmak, Turkey)	Hydrogel (without any chemicals or crosslinking agents)	Viscosity ranged from 49,709 to 10,219 cP	FTIR peaks at 2910 cm^−1^, 1650 cm^−1^, 1054 cm^−1^, and 3300 cm^−1^	Salva et al. (2023) [96]
Common type of honey (Yazd, Iran)	Hydrogel film (freeze–thaw method)	Swelling ratio ranged from 324 ± 18% to 421 ± 11% Weight loss ranged from 52.5 ± 4.8% to 57.7 ± 8.8% Tensile strength ranged from 16.7 ± 0.3 to 19.8 ± 4.8 Mpa Elongation break varied from 241.0 ± 64.5% to 421.6 ± 45.8%	DSC thermograms (thermal behaviour) revealed 3 endothermic peaks at 168 °C, 232 °C, and 258 °C	Koosha et al. (2021) [100]
Manuka honey	Gas-blown porous hydrogel sheets (redox-initiated free radical)	Swelling percentage of the hydrogels ranged from 1100% to 2000% and from 3100% to 4100% for 1% and 10% Manuka honey-containing hydrogel, respectively Porosity was of 21.73 ± 15.5% and 42.22 ± 11.1 for 1% and 10% Manuka honey-containing hydrogel, respectively	SEM revealed that the average pore sizes were 50.5 ± 11.2 µm and 51.5 ± 24.2 µm	Pinthong et al. (2023) [101]
Manuka honey	3D bioprinting hydrogel	The water uptake ability reached 500 ± 60% 3D bioprinting spreading ratio was 3.5 ± 0.1 mm, ranging from 0.5 mm as it exited the nozzle to 1.0 mm at the bottom The extrusion of longer fibres (13 mm) than bioink without honey, which formed a shorter, droplet-shaped filament (7 mm)	SEM revealed that the average pore sizes were 87.2 ± 9.5 µm	Scalzone et al. (2022) [116]
Manuka honey	3D bioprinting hydrogel	Swelling ratio of 3D bioprinting hydrogel was ranged from 400 to 480% for Manuka honey/pectin ratios below 0.025 (*w*/*w*) and above 0.1 (*w*/*w*) Minimal shape deviation from the theoretical dimensions of the patches when pectin concentrations ranged from 0.125 to 0.18% (*w*/*v*) in the ink and the Manuka honey/pectin ratio was above 0.05 (*w*/*w*)	Not stated	Andriotis et al. (2020) [104]
Natural honey	3D bioprinting hydrogel	The weight loss ranged from 15 to 20%	FTIR peaks at 3372 and 3264 cm^−1^	Hu et al. (2024) [111]
Raw honey (Layezangan, Fars, Iran)	Hydrogel (inter-polyelectrolyte complex method)	WVTR at 380.4 ± 21.5004 g/m^2^/day Contact angle at θ = 87.3° Porosity was 53.28% and 60.29%	Not stated	Saberian et al. (2021) [108]

**Table 2:** Studies on the use of honey in biomaterials and scaffolds in vitro and in vivo for therapeutic applications.

Types of Honey	Scaffold Type	Biomaterials	Antibacterial Effects	Biocompatibility and Cell Proliferation	Animal Model and Healing Effects	References
Manuka Honey	Hydrogel	Chitosan, polyvinyl alcohol (PVA), honey	Antibacterial effects against *S. aureus* and *E. coli*	Not stated	Not stated	Chopra et al. (2022) [106]
Stingless Bee Honey, Giant Bee Honey, and Asian Bee Honey	Hydrogel	Sodium carboxymethyl cellulose (SCMC), hydroxypropyl methyl cellulose (HPMC), polyethylene glycol (PEG), honey	Antibacterial effects against *S. aureus* and *E. coli*	Human skin fibroblast cells: MTT assay and cell scratch assay	Not stated	Gopal et al. (2021) [37]
Stingless Bee Honey	Hydrogel	Sodium carboxymethyl cellulose (SCMC), hydroxypropyl methyl cellulose (HPMC), polyethylene glycol (PEG), poly(lactic-co-glycolic acid) (PLGA), honey	Antibacterial effects against *S. aureus* and *E. coli*	Human skin fibroblast cells: MTT assay and cell scratch assay	Not stated	Lo et al. (2021) [123]
Manuka Honey	Electrospun Nanofibers	Polyvinyl alcohol (PVA), pomegranate peel extract, bee venom, honey	Antibacterial effects against *S. aureus* and *E. coli*	L929 mouse fibroblast cells: MTT assay	Adult female Sprague Dawley rats with excisional wounds of 45 mm^2^ and healing duration of 10 days for all honey-containing hydrogels	Zekry et al. (2020) [124]
Stingless Bee Honey	Electrospun Nanofibers	Gelatin, curcumin, honey	Antibacterial effects against *S. aureus*, *E. coli*, *K. pneumonia*, *MRSA*, *P. aeruginosa*, and *A. baumannii*	L929 mouse fibroblast cells: wound scratch assay	Male Wistar albino rats with excisional wounds of 314 mm^2^ and healing duration of 17 days for the honey-containing nanofibrous membrane	Samraj et al. (2020) [125]
Manuka Honey	Hydrogel	Gellan gum, inorganic clay, honey	Antibacterial effects against *S. epidermidis* and *S. aureus*	Human mesenchymal stem cell (hMSC): trypan blue exclusion test, PrestoBlue™ assay and DAPI staining and fluorescence microscopy	Adult wild-type mice (C57BL/6JOlaHsd) with excisional wounds of 16 mm^2^ started to show immune responses and antibacterial effects after 1 week	Bonifacio et al. (2020) [126]
Dabur Honey	Hydrogel	Sodium alginate, honey	Antibacterial effects against *MRSA* and *E. coli*	Human dermal fibroblast and human epidermal keratinocytes: MTT assay	Pathogen-free male Wistar rats with excisional wounds of 1 cm in diameter showing rapid healing on day 8 and fully closed by day 12 for 4% honey concentration	Mukhopadhyay et al. (2020) [103]
Manuka Honey	Electrospun Fibrous Mat	Poly(ϵ-caprolactone) (PCL), methylcellulose (MC), honey	No antibacterial effects against *S. aureus* and *E. coli*	Human dermal fibroblasts (hDFs) and HaCaT cells: WST-8-assay and cell scratch assay	Not stated	Schuhladen et al. (2020) [127]
Raw and unprocessed honey	Electrospun Nanofibrous Mat	Polyvinyl alcohol (PVA), cellulose acetate (CA), curcumin, honey	Antibacterial effects against *E. coli*	Not stated	Not stated	Gaydhane et al. (2020) [128]
Manuka Honey	Hydrogel	Gellan gum (GG), virgin coconut oil (VCO), honey	Not stated	Not stated	Six-week-old female Sprague Dawley rats with excisional wound diameters of 8 mm showing clear epidermal regeneration after 14 days for 20% honey concentration	Iryani et al. [130]
Natural Honey	Hydrogel	Polyvinyl alcohol (PVA), chitosan, clay, honey	Antibacterial effects against *S. aureus*	Peripheral blood mononuclear cells: MTT assay	Female Syrian mice with excisional wounds of 100 mm^2^ showing complete healing for honey-containing hydrogel at day 12	Noori et al. (2018) [36]
Manuka Honey	Hydrogel	Chitosan, Carbopol 934, honey	Antibacterial effects against *P. aeruginosa*, *S. aureus, K. pneumonia,* and *S. pyogenes*	Not stated	Eight-week-old albino mice with excisional wounds of 10 mm in diameter showing near-complete healing at day 9 for 75% honey concentration	El-Kased et al. (2017) [97]
Natural Honey	Hydrogel	Polyvinyl alcohol (PVA), egg white, clay nanoparticles, honey	Not stated	Human peripheral blood mononuclear cells (PBMCs): flow cytometry assay	Eight-week-old female BALB/c mice with excisional wounds in the deep fascia showing near-complete healing at day 10 for bionanocomposite hydrogel group	Rafati et al. (2019) [131]
Raw Honey	Hydrogel	Polyvinyl alcohol (PVA), chitosan, gelatin, honey	Antibacterial effects against *P. aeruginosa* and *S. aureus*	Human skin fibroblast cells: MTT assay	Male Wistar rats with excisional wounds of 4 cm^2^ showing a higher number of fibroblast cells in the epidermal layer on day 20 for the group with 20% honey concentration	Shamloo et al. (2021) [99]

## Data Availability

No new data were created or analyzed in this study.

## Abbreviations

The following abbreviations are used in this manuscript:

WVTR	Water Vapour Transmission Rate
FTIR	Fourier Transform Infrared Spectroscopy
SEM	Scanning Electron Microscopy
ROS	Reactive Oxygen Species
3D	Three-Dimensional
2D	Two-Dimensional
H_2_O_2_	Hydrogen Peroxide
GOx	Glucose Oxidase
NASAIDs	Non-steroidal anti-inflammatory medications
IL-6	Interleukin 6
TNF-α	Tumour Necrosis Factor-alpha
NO	Nitric Oxide
COX-2	Cyclooxygenase-2
COX-1	Cyclooxygenase-1
PGE2	Prostaglandin E2
NF-κB	Nuclear Factor kappa-light-chain-enhancer of activated B cells
IκBα	Inhibitor of Nuclear Factor kappa B alpha
PAI	Plasminogen activator inhibitor
NH_2_	Amino group
COOH	Carboxylic acid group
OH	Hydroxyl group
CONH_2_	Amide group
CONH	Secondary amide group
SO_3_H	Sulfonic acid group
UV	Ultraviolet
N-H	Nitrogen–Hydrogen Bond
F-H	Fluorine–Hydrogen Bond
DPBS	Dulbecco’s Phosphate-Buffered Saline
PBS	Phosphate-Buffered Saline
C-H	Carbon–Hydrogen Bond
C=O	Carbonyl Group
C-O	Carbon–Oxygen Bond
HaCaT	Human Adult Low Calcium High Temperature Keratinocytes
